# Individualised Timing of Radio-Guided Parathyroidectomy Using Multi-Phase SPECT/CT Increases In Vivo Sensitivity and Accuracy and Reduces Operating Time: A Randomised Clinical Trial

**DOI:** 10.3390/diagnostics11040677

**Published:** 2021-04-09

**Authors:** Martin Formánek, Vladimír Dedek, Michal Koláček, Martin Havel, Karol Zeleník, Pavel Komínek

**Affiliations:** 1 Department of Otorhinolaryngology and Head and Neck Surgery, University Hospital Ostrava, 17. Listopadu 1790, 70852 Ostrava, Czech Republic; karol.zelenik@fno.cz (K.Z.); pavel.kominek@fno.cz (P.K.); 2 Clinic of Nuclear Medicine, University Hospital Ostrava, 17. Listopadu 1790, 70852 Ostrava, Czech Republic; vladimir.dedek@fno.cz (V.D.); michal.kolacek@fno.cz (M.K.); martin.havel@fno.cz (M.H.)

**Keywords:** minimally invasive parathyroidectomy, individualised timing, multi-phase SPECT/CT, superior in vivo results

## Abstract

**Background**: Minimally invasive parathyroidectomy is the preferred treatment for primary hyperparathyroidism. Despite relatively accurate preoperative information, minimally invasive parathyroidectomy can be challenging, especially in the case of small and ectopic adenomas. Radio guidance aids in both in vivo identification and ex vivo confirmation of adenoma. In vivo accuracy is currently not satisfactory. The present study evaluated whether a beneficial effect (increased sensitivity, specificity, accuracy) is obtained with individualised timing of minimally invasive radio-guided parathyroidectomy (MIRGP) using preoperative multi-phase 99mTc-MIBI single photon emission computed tomography (SPECT)/computed tomography (CT). **Methods:** This randomised clinical trial was conducted from May 2016 to January 2020 in a tertiary referral hospital. Adult patients with primary hyperparathyroidism sent for 99mTc-MIBI SPECT/CT were included consecutively and randomly assigned to conventional (dual-phase) SPECT/CT and conventional MIRGP (group I) or multi-phase SPECT/CT and individualised MIRGP (group II). One hundred of 106 eligible patients were included, and 83 patients underwent complete intervention. **Results:** A total of 47 patients in group I and 35 patients in group II were analysed. Group II had a shorter operating time (*p* = 0.003). The in vivo sensitivity and accuracy of radio guidance was 85.1% in group I and 100% in group II (*p* = 0.046), and 90.4% in group I and 100% in group II (*p* = 0.021), respectively. We found no difference in the in vivo specificity and ex vivo parameters between groups. **Conclusion:** Individualised timing increased the in vivo sensitivity and accuracy of radio guidance and reduced operating time, as some parathyroid adenomas rapidly wash out the radionuclide.

## 1. Introduction

Primary hyperparathyroidism is a common endocrine disorder with approximately 100,000 new cases diagnosed each year in the United States [1,2]. Surgical removal is the only causal treatment [2]. Minimally invasive parathyroidectomy (MIP) is currently the approach of choice, as it allows the possibility of local anaesthesia and same-day discharge. The procedure also limits the size of the incision (2–2.5 cm), reducing operating time, wound healing time, postoperative pain, analgesic request rate and consumption, risk of infection, and risk of post-surgical hypocalcaemia, and providing significantly better cosmetic results than the standard procedure with bilateral cervical exploration [3,4,5].

MIP was made possible by the introduction of technetium-99m methoxyisobutylisonitril (99mTc-MIBI) scintigraphy for preoperative localisation of parathyroid adenomas. The detection rate of 99mTc-MIBI single photon emission computed tomography (SPECT)/CT currently reaches 88–89% [6,7]. SPECT/CT has equivalent sensitivity as SPECT but provides superior topographic information [8].

Despite relatively accurate preoperative topographic information, MIP can still be very challenging, especially in the case of small ectopic adenomas (e.g., the anterior mediastinum, the retroesophageal region), which are found in 22% of cases with primary hyperparathyroidism [7]. In addition, even the best imaging modalities sometimes fail to identify lesions accurately.

The success rate of MIP can be improved by frozen section analysis, endoscopy, intraoperative parathyroid hormone (PTH) monitoring, radio-guided techniques, or combinations thereof [3]. Each method has its advantages. However, radio guidance is the only technique that offers help with both in vivo identification of the adenoma and ex vivo confirmation of adenoma removal [9,10]. Ex vivo confirmation is possible with shorter delay than in the case of intraoperative, post-excision PTH measurement [10]. The cure should be indicated by gamma probe readings showing that the resected gland emits radioactive counts >20% of background, the so-called Norman 20% rule [9,11,12]. Adenomas and hyperplastic glands usually present ex vivo counts well over 20% of background [13,14,15,16]. However, at least half of a parathyroid gland must be resected to reliably identify a hyperfunctional lesion [13]. Many authors agree that compliance with these criteria results in near 100% ex vivo sensitivity and specificity and allows successful minimally invasive radio-guided parathyroidectomy (MIRGP) in up to 97–99% of positive SPECT/CT cases [9,11,12,13,14,16].

However, ex vivo accuracy does not help a surgeon with the most demanding part, as this measurement is obviously only possible after the adenoma has already been found and excised. Therefore, in vivo accuracy is most important, especially in the case of small ectopic adenoma. In vivo sensitivity of radio guidance only reaches 87–93%, with a positive predictive value of 88% and accuracy of 83% [10,14]. In the rest of cases, it does not help the surgeon find the adenoma, or is even misleading [14].

The aim of the present study was to determine whether there is a beneficial effect on in vivo parameters (increased sensitivity, specificity, accuracy) of radio guidance during MIP from individualised timing of surgery using preoperative multi-phase 99mTc-MIBI SPECT/CT.

## 2. Methods

### 2.1. Ethical Considerations

This prospective study was approved by the ethics committee of the university hospital and performed following the Declaration of Helsinki according to good clinical practice and applicable regulatory requirements. The study was registered at ClincialTrials.gov (accessed on 3 February 2021) (NCT04344886). Written informed consent was obtained from each patient before initiating any procedure.

### 2.2. Design and Setting

The randomised clinical trial was conducted from May 2016 to January 2020 in a tertiary referral hospital.

### 2.3. Participants and Randomisation

Adult patients with no history of thyroid or parathyroid surgery who had been diagnosed with primary hyperparathyroidism and sent for 99mTc-MIBI SPECT/CT at a tertiary referral hospital were consecutively included in the study. Using random number generation, patients were assigned to conventional (dual-phase) SPECT/CT and conventional MIRGP (group I) or multi-phase SPECT/CT and individualised MIRGP (group II). The power analysis was performed to estimate the sample size with the power 80% and the level of significance 5%. Firstly, the power analysis was performed with the expected accuracy 83% in Group I and 99.9% in Group II [14]. The estimated minimum sample size was 62 patients (31 patients in each group). Then, the power analysis was performed with the expected sensitivity 87% in Group I and 99.9% in Group II [10]. The estimated sample size was 86 patients (43 patients in each group). Therefore, we decided to include 100 patients (50 patients in each group) considering patient exclusion.

**Figure 1:**
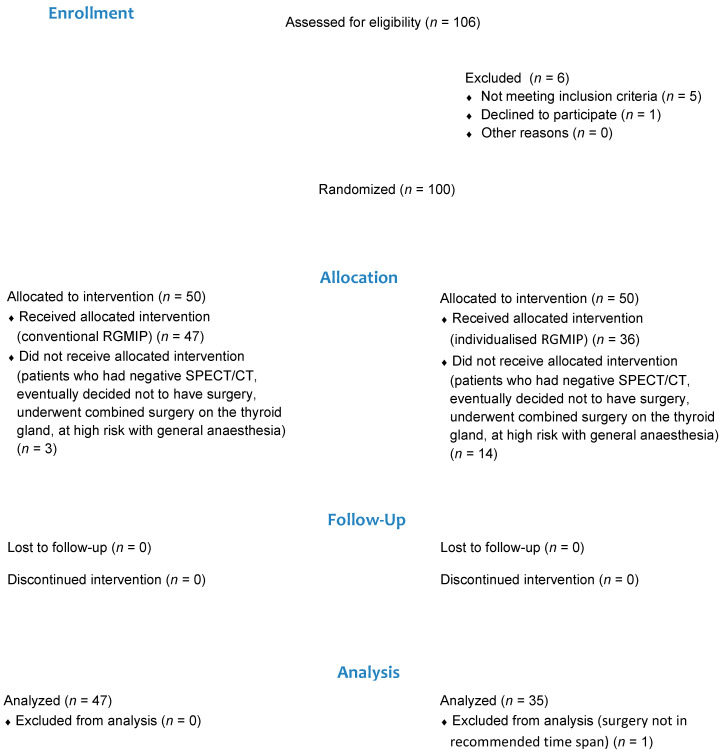
Flow diagram of inclusion in the clinical trial.

One hundred of 106 eligible patients were included in the study and randomised; 83 patients received MIRGP (Figure 1). Patients who had negative SPECT/CT, eventually decided not to have surgery, underwent combined surgery on the thyroid gland, at high risk with general anaesthesia, or did not undergo MIRGP in the recommended time span after radionuclide administration were excluded from the study. Absolutely dominant reasons for not undergoing whole intervention were combined surgery on the thyroid gland and decision not to have a surgery. Negative SPECT/CT was present in one patient in both groups.

### 2.4. SPECT/CT

SPECT/CT was performed using a Symbia-Intevo eXcel scanner (Siemens Healthcare GmbH, Erlangen, Germany) with the following acquisition parameters: step and shoot mode, matrix 256 × 256, pixels 1.65 × 1.65 mm, zoom 1.45, 64 views, time per view 20 s. Low-dose CT was always performed.

#### 2.4.1. Group I

A 600 MBq 99mTc-MIBI intravenous administration was performed in group I. Detection was performed 10 and 150 min after application. Examination was performed visually by a nuclear medicine specialist regarding localisation of the adenoma. Standard iterative reconstruction parameters were used with the Flash3D algorithm.

#### 2.4.2. Group II

A 600 MBq 99mTc-MIBI intravenous administration was performed in group II. The exact amount of radioactivity in the syringe to an accuracy of 1 MBq was measured immediately prior to and after administration (“syringe before” and “syringe after”, respectively) and entered into the acquisition program. The patient’s weight was also entered. Detection was performed after 10, 90, 150 and 210 min. Detection after 210 min was indicated only if there was still uptake in the parathyroid gland after 150 min. Examination was performed visually by a clinician regarding localisation of the adenoma. In addition, the examination was processed using xSPECT Quant software (Siemens). Unlike classic SPECT reconstruction, xQuant uses the ordered subset conjugate gradient minimisation (OSCGM) algorithm. The volumes of interest were marked (pathological parathyroid gland and contralateral thyroidal lobe) and the standardised uptake value (SUV) calculated. Thereafter, an individual time after preoperative administration was recommended for every patient based on change in SUV with time.

#### 2.4.3. Surgery

Before surgery, 600 MBq of 99mTc-MIBI was administered intravenously to each patient. An excision was made between 2 and 3.5 h after administration in group I and at the recommended time after administration in group II. All surgeries were performed under general anaesthesia. A typical incision 2.5 cm in length was made in the region of the suspected adenoma. Intraoperative scanning for radionuclide counts more than background was performed to localise abnormal parathyroid gland(s). The background measurement was taken just over the thyroidal isthmus before excision of the pathological gland. We refer to these measurements as in vivo counting. The in vivo index, defined as the ratio of in vivo counting to background (both expressed in counts per second), was used as an operational quantity. The parathyroid tissue was considered pathological when the in vivo counting was at least 1.15-times more than the background. Radioactive ex vivo count in the adenoma/hyperplastic parathyroid gland greater than 20% of background was used as the cut-off for cure. The background measurement was taken just over the thyroidal isthmus after excision of the pathological gland. A wireless hand-held gamma probe detector, EuroProbe3 (Capintec, Inc., Florham Park, NJ, USA) with 5 mm diameter, was used. All surgeries were performed by an experienced neck surgeon.

#### 2.4.4. Follow-Up

Serum levels of parathyroid hormone were determined one day after surgery. Calcaemia was monitored daily for seven postoperative days. Patients were followed for three weeks until the result of their bioptic specimen were available. The specimen was evaluated in the same centre.

#### 2.4.5. Statistical Analysis

The primary analysis was performed using standard tools of exploratory data analysis. For the description of numerical variables, the mean and standard deviation (SD) or median and interquartile range (IQR) were used. Categorical variables are presented with absolute and/or relative frequencies. Observed groups were compared in terms of demographics (e.g., age, sex), the presence of possible risk factors (e.g., ectopic adenoma, multiple adenomas), and treatment and detection results (e.g., in vivo and ex vivo sensitivity, specificity, treatment success rate, operating time, time at which adenoma was excised, volume of adenoma).

Non-parathyroid or non-pathological parathyroid tissues for which uptake reached a significant count are considered false-positives. Cases in which the uptake of the pathological parathyroid gland did not reach a significant count were classified as false negatives. Surgery was considered successful if serum levels of parathyroid hormone and calcaemia decreased to normal with histological confirmation of parathyroid gland adenoma/hyperplasia. Each adenoma was evaluated separately in cases of multiple adenomas.

The statistical analysis was performed using R software (version 3.6.0). The following statistical tests were used: Mann–Whitney *U*-test, Fisher’s exact test, Spearman´s correlations with test of significance, and test for the homogeneity of two binomial proportions with a 5% significance level.

## 3. Results

A total of 83 patients underwent MIRGP, 47 in group I (conventional MIRGP) and 36 in group II (individualised MIRGP). One patient in group II had to be excluded from the analysis because surgery was not performed in the recommended time span after radionuclide administration. Therefore, 47 patients in group I and 35 patients in group II were analysed. Compliance with follow-up was 100%. No differences were found between the groups in regards to age, sex, or presence of ectopic adenomas (Table 1).

**Figure 2:**
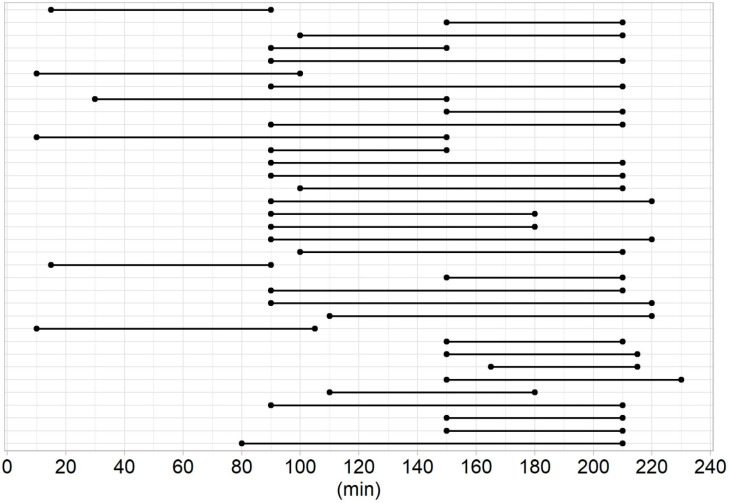
Visualisation of the recommended time span for surgery from radionuclide administration in every patient in group II.

**Figure 3:**
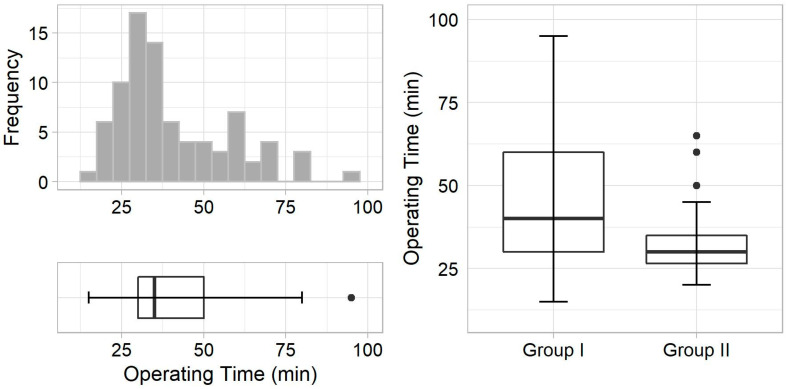
(**Left**)—Operating time frequency (**upper**) and distribution (**bottom**) in all patients in both groups. (**Right**), comparison of operating time distribution between two groups.

Surgery was successful in 98% of patients in group I and in 100% of patients in group II. A lymph node was excised instead of parathyroid adenoma in one case in group I. Seven rapidly washed-out adenomas were identified in group II, the removal of which was never recommended after 150 min of radionuclide administration (Figure 2). Group II had a significantly shorter operating time, and tended to have a lower adenoma volume and longer time delay than group I (Figure 3 and Table 2). We found no difference in histology, presence of multiple adenomas, adenoma and background count rate, in vivo index value, or number of complications. A weak to moderate negative correlation between the adenoma count rate and time delay, and background count rate and time delay was observed in both groups. No other correlation was found.

Seven false-negative cases and two false-positive cases were found in group I but none in group II. The in vivo sensitivity and accuracy of radio guidance was significantly higher in group II than group I. We found no difference in in vivo specificity and ex vivo parameters between the two groups (Table 3).

## 4. Discussion

Radio guidance is often used in MIP due to its advantages over other methods; it offers excellent ex vivo results with the 20% rule [9,12,13,14,16]. The results are fully comparable to intraoperative PTH monitoring, which has 99% sensitivity, 98% accuracy, and a positive predictive value of 99.6% [14]. In addition, ex vivo confirmation is possible almost immediately. The only question now is whether radio guidance is able to reliably ex vivo differentiate adenoma from hyperplastic gland [11,13,15]. Excellent ex vivo radio guidance results were confirmed in all patients in the present study, but the in vivo properties are most important.

In a previous study of 845 patients with primary hyperparathyroidism, the main cause of operative failure of MIP using PTH monitoring was the surgeon’s inability to find the abnormal parathyroid gland [17]. Unfortunately, the in vivo radio guidance results are still not satisfactory. These results are unlike ex vivo results rarely reported. Chan et al. reported that in vivo radio guidance achieved a sensitivity of 93%, positive predictive value of 88% and accuracy of 83% [14]. García-Talavera et al. reported 87% sensitivity, 95% specificity and a positive predictive value of 97% when using a cut-off value of 1.15 for the gamma probe in vivo index, and 67% sensitivity, 87% specificity and a positive predictive value of 95% when using a cut-off value of 1.51 [10]. Thus, a risk of both false positives and false negatives has been reported.

Cases in which uptake of non-parathyroidal or non-pathological parathyroid tissues reached a significant count are labelled as false-positives. It can be caused by, for example, concomitant thyroid disease, such as an undiagnosed follicular thyroid adenoma. Nevertheless, these cases are rare and usually apparent on gross examination by the operating surgeon [18]. In the present study, only two such cases were observed.

Cases in which radio guidance does not identify parathyroid adenoma are labelled as false-negatives and are generally thought to be caused by too long of a delay in surgery from administration of the radionuclide [10]. The principle underlying scintigraphy and MIRGP is that administered radionuclide accumulates in both thyroid and pathological parathyroid tissue. The radionuclide is washed out from the thyroid tissue relatively quickly, and from parathyroid adenoma slowly, allowing its localisation [19]. However, every adenoma acts (and washes out radionuclide) differently based on its volume and number of mitochondria-rich oxyphil cells [20]. The time from administration of the radionuclide is a parameter for which the greatest inconsistency is observed between studies and between individual MIRGP procedures in one study. Norman’s group originally proposed performing surgery within 3.5 h from scintigraphy, as all 345 surgeries in their study were performed 1.5–3.5 h from administration of the radionuclide [11]. In his later studies, he tried to perform MIRGP as early after administration of the radionuclide as possible, performing 5000 MIRGP procedures within 2.25 h, with approximately 90% of operations performed between 40 min and 1.5 h [21]. The studies by Chen et al. and García-Talavera et al. reported performing MIRGP 1–2 h and 30 min–3 h after radionuclide administration, respectively [10,14].

Basically, there is only one rule based on Norman´s empiric results: surgery should be performed within 3.5 h [11]. The authors of the present study considered a wide, non-individualised time span from radionuclide administration too robust and the main cause of unsatisfactory in vivo MIRGP results. The peak time for visualisation varies among individuals. Based on the data from the literature and clinical diagnostic scintigraphy experience, a considerable number of adenomas rapidly wash out the radionuclide, almost imitating the thyroid gland [20]. Therefore, if rapid wash-out of adenoma occurs and the time span of surgery is at the upper limit of the range, the in vivo difference between adenoma and background will be too small to reach significance and to guide the surgeon. The results of our study confirm this theory. We even found adenomas in which extirpation within 15–90 min was recommended based on the calculated SUVs. On the other hand, there was also adenoma in which the recommended time span ended at 230 min. The specific time at which all recommendations would be met was not observed. This clearly indicates a complexity of the whole issue and advocates a necessity for an individualised setting. The exact time at which there is the greatest difference between adenoma and background activity was identified by the SUV calculation. We were able to achieve significant 100% in vivo sensitivity and accuracy using the cut-off value of 1.15 for the gamma probe index and significantly reduced the operating time for MIRGP. The results directly show how much the probe benefited the surgeon. In addition, it minimised radiation exposure for surgeons and operating room personnel. The results were achieved even with the insignificantly smaller adenoma volume and the same number of ectopic adenomas. Notably, the reduced operating time is also relative to MIRGP, only in a different setting. Operating time would be even more significant when compared to intraoperative PTH monitoring. Importantly, a collimated gamma probe was used, which allows minimisation of interfering radioactivity from the surrounding tissues [22]. The authors believe that 100% in vivo sensitivity was possible only because exclusively SPECT/CT-positive patients were included. Based on recent data, we could expect that all in vivo and ex vivo parameters would be worse in both groups if patients with negative SPECT/CT were also included [23].

Surprisingly, individualised treatment also made logistics easier. The time span at which surgery is recommended is known several days to weeks in advance. It is also known in which cases it is necessary to “hurry” after radionuclide administration, and in which cases there is relatively more time. Because adenomas that rapidly wash out radionuclide are a minority, personnel are not overloaded as they would be if each case had to be dealt with as soon as possible, as was the case in Norman’s study [21]. This is visible in the results when the time delay from radionuclide administration was higher, though insignificantly, in group II. In several cases the recommendation was even to perform surgery later than at 3.5 h.

The most common surgical complication in a series of 1037 MIPs was recurrent nerve injury in 0.77% [5]. Our data correspond with these results and with the results of other high-volume hospitals [24]. Therefore, it is reasonable to consider MIP safe [24].

The main disadvantage of individualised treatment is that the preoperative phase is more time-consuming, as one or two extra scans equates to 22–44 extra minutes in our setting. Extra scanning also means additional radiation for patients from CT; nevertheless, only the extra low-dose setting was used, resulting in an effective dose of approximately 1.7 mSV from all four CT scans. This is a minimal dose increase considering the total effective dose of approximately 14.4 mSv from two radionuclide administrations.

## 5. Conclusions

Individualised timing for MIRGP was possible using preoperative multi-phase SPECT/CT. Individualised timing significantly increased the in vivo sensitivity and accuracy of radio guidance up to 100%, and also significantly reduced operating time, with all its associated benefits. Therefore, individualised timing should be routinely recommended.

## Figures and Tables

**Table 1:** Characteristics of the study participants.

Variable	Group I (*n* = 47)	Group II (*n* = 35)	*p*-Value
Age, years	64.0 (55.0–71.0)	62.0 (53.0–67.5)	0.183 *
Females	34 (72)	27 (77)	0.799 **
Males	13 (28)	8 (23)	
Ectopic adenoma	17 (36)	12 (34)	>0.999 **

Data are presented as the median (interquartile range) or *n* (%). * Mann–Whitney test; ** Fisher exact test.

**Table 2:** Comparison of results between groups.

Result	Group I (*n* = 47)	Group II (*n* = 35)	*p*-Value
Successful surgery	46 (98)	35 (100)	>0.999 *
Histology, hyperplasia	1 (2)	1 (3)	>0.999 *
Histology, adenoma	45 (96)	34 (97)	>0.999 *
Multiple adenomas	1 (2)	1 (3)	>0.999 *
Time delay, min	160 (125–190)	190 (140–205)	0.260 **
Parathyroid gland volume, mL	1.8 (1.0–3.0)	1.3 (1.0–2.4)	0.234 **
Operating time, min	40.0 (30.0–60.0)	30.0 (26.5–35.0)	0.003 **
Adenoma count rate, cpm	1200 (860–1400)	1200 (1100–1405)	0.318 **
Background count rate, cpm	700 (500–800)	720 (600–850)	0.094 **
In vivo index	2.0 (1.5–2.2)	1.8 (1.4–2.0)	0.285 **
Recurrent laryngeal nerve paralysis	1 (2)	0 (0)	>0.999 *
Hypocalcaemia	2 (4)	1 (3)	>0.999 *

Data are presented as the median (interquartile range) or *n* (%). * Fisher exact test; ** Mann–Whitney test.

**Table 3:** Comparison of in vivo and ex vivo parameters of radio guidance between groups.

Parameter	Group I	Group II	*p*-Value *
In vivo sensitivity	40/47 (85.1%)	35/35 (100%)	0.047
In vivo specificity	45/47 (95.7%)	35/35 (100%)	0.609
In vivo accuracy	85/94 (90.4%)	70/70 (100%)	0.021
Ex vivo sensitivity	47/47 (100%)	35/35 (100%)	>0.999
Ex vivo specificity	47/47 (100%)	35/35 (100%)	>0.999
Ex vivo accuracy	94/94 (100%)	70/70 (100%)	>0.999

Data are presented as *n*/*n* (%). * Test for the homogeneity of two binomial proportions.

## Data Availability

All relevant data are presented.
